# Are Emotional and Behavioral Problems of Infants and Children Aged Younger Than 7 Years Related to Screen Time Exposure During the Coronavirus Disease 2019 Confinement? An Exploratory Study in Portugal

**DOI:** 10.3389/fpsyg.2021.590279

**Published:** 2021-02-26

**Authors:** Rita Monteiro, Nuno Barbosa Rocha, Sandra Fernandes

**Affiliations:** ^1^Ph.D. Program in Educational and Behavioral Sciences, University of Vigo, Vigo, Spain; ^2^Center for Rehabilitation Research, School of Health, Polytechnic Institute of Porto, Porto, Portugal; ^3^Portucalense Institute for Human Development (INPP), Department of Psychology and Education, Portucalense University, Porto, Portugal

**Keywords:** COVID-19 confinement, children’s well-being, screen time, digital media, emotional problems, behavioral problems

## Abstract

The coronavirus disease 2019 outbreak forced most of the world’s population to be confined at home to prevent contagion. Research reveals that one of the consequences of this confinement for children is an increased amount of time spent using screens (television, computers, and mobile devices, etc.) at home. This exploratory study aims to analyze the association between screen time exposure and emotional/behavioral problems of infants and children aged under 7 years, as manifested during the lockdown period in Portugal due to the coronavirus disease 2019 outbreak. The study was controlled for sociodemographic and confinement variables. A sample of 193 parents of children aged from 6 months to 6 years and 12 months, residing in Portugal, completed a survey about the time and manner of use of screen time exposure of their children. Data were derived on circumstances both before and after the confinement; the survey also explored the child’s behavioral and emotional adjustment. The findings revealed a modest relationship between children’s exposure time to screens and behavioral and emotional problems on children studied. It was also found that parents may play an important role in children’s behavioral and emotional adjustment during the confinement period.

## Introduction

Originating in the City of Wuhan (China), in December 2019, the coronavirus disease 2019 (COVID-19) outbreak emerged and quickly disseminated throughout the world. At the end of April, in Portugal, about 24,692 cases were confirmed and 989 deaths reported (Direção_Geral_de_Saúde, 2020a), rising to 42,171 and 1,576, respectively, at the end of June (Direção_Geral_de_Saúde, 2020b). On March 13, the Portuguese government instituted emergency measures forcing the population to be confined in their residences for a long period. This brought several changes to daily routines: schools were closed, and children started to have classes remotely at home; many workers lost their jobs or had to stay at home working remotely. It was the first time in Portuguese history that quarantine was implemented to control a pandemic.

It is known that the psychological impact of quarantining is wide-ranging, substantial, and can be long-lasting (Brooks et al., 2020). Despite limited evidence on the impact of confinement on children’s mental health, surveys of students in China showed an increase in anxiety (Cao et al., 2020; Xie et al., 2020) and depression (Xie et al., 2020). Results of a study developed in Italy and Spain to examine the impact of the COVID-19 quarantine on children and adolescents (Orgilés et al., 2020) showed that 85.7% of the parents reported negative changes in their children’s emotional state and behaviors. The main symptoms were difficulty in concentrating, boredom, irritability, restlessness, nervousness, and feelings of loneliness; being more uneasy and more worried were also reported by the parents. Over a 1-month lockdown period, parents or caregivers of primary-school-age children reported an increase in their child’s emotional, behavioral, and restlessness/attentional difficulties (Pearcey et al., 2020a).

Several factors may contribute to this. The reduction of social interaction and the reduction of outdoor activities (Xie et al., 2020) and even access to outside space (Pearcey et al., 2020b) seem to play an important role. Factors related to the place where the children live, financial stability of parents, concerns about the pandemic situation, or the existence of family members diagnosed with COVID also have an impact on emotional well-being (Cao et al., 2020). Furthermore, children’s well-being seems directly related to the level of stress and well-being of parents (Orgilés et al., 2020), which can be influenced by factors related to the economic and family situation, concerns about the severity of the pandemic situation, proximity to infected people and home conditions such as the dimensions of the house or the existence or not of an open-air space (Rodríguez-Rey et al., 2020).

Due to COVID-19, most children in Organization for Economic Cooperation and Development (OECD) countries were more exposed to digital technologies than usual (OECD, 2020). Digital tools take on an important role in providing tools for children, parents, and teachers to continue schooling, facilitate social interactions, and provide recreational activities and psychological and social support from outside. On the other hand, widespread digitalization has its downsides, including increasing exposure to hateful, harmful, or illegal content and cyberbullying (OECD, 2020). In Spain and Italy, during the quarantine, children spent more time using screens (iPads, TVs, mobiles, or computers), slept more hours, and spent less time doing physical activity (Orgilés et al., 2020). This is concerning insofar as there are pieces of evidence that a higher screen-time exposure is correlated with greater sedentary time and lack of physical activity on children, which, in turn, increases the risk of psychological problems (Hamer et al., 2009; Pagani et al., 2010; Page et al., 2010). By itself, confinement leads to a decrease in physical activity, which, in interaction with increased screen-time exposure, may lead children to develop more emotional and behavioral problems.

Studies about the impact of exposure to the new technologies are still controversial. Some research (Radesky et al., 2015) reveals the benefits of children’s exposure to digital media with respect to the development of comprehensive and cognitive skills. On the other hand, other studies have found an array of negative outcomes due to children’s exposure to media: cognitive and language delays (Zimmerman and Christakis, 2005; Zimmerman et al., 2007), nutritional and physical problems, and aggressiveness (Jordan, 2004). Existing evidence showed a negative association between screen-time and psychological well-being among children and adolescents (Twenge and Campbell, 2018; Zhao et al., 2018).

Despite being described in the literature that there is a decrease in children’s well-being during a period of confinement and an increase in the time of exposure to the screens, we have not found any study that intends to test if there is a relationship between screen exposure and behavioral and emotional problems during a confinement situation. Therefore, the main objective of this exploratory study was to analyze the association between screen-time exposure during the lockdown period in Portugal due to the COVID-19 outbreak and the emotional and behavioral problems of infants and children aged younger than 7 years old (while looking for the contribution of sociodemographic and confinement-related variables). Specifically, we aimed to know: the time and conditions of exposure to screens; parents’ perception of children’s exposure time to screens; conditions related to confinement, considering it as the independent variable when studying its relationship with children’s emotional and behavioral state during the confinement period (dependent variable). We hypothesized that higher screen-time exposure during the lockdown would lead children to have higher risks of emotional and behavioral problems. We also hypothesized that confinement conditions (personal situation or home features) might have an influence on children’s emotional and behavioral states.

## Materials and Methods

### Participants

To reach the largest number of people, the survey was released online and shared through the personal contacts of the research team, through kindergarten schools and social media groups associated with families and parenting in Portugal. The survey was disseminated in a snowball manner in which participants could reach other participants. We did not control the dissemination within the kindergarten schools. The participants in this study were parents or caregivers (hereafter referred to as “parents”) of the children. To participate, parents had to be aged 18 years or older, caring for a 6-month to a 6-year-and-12-month-old child, and self-reporting as being literate in Portuguese. Children were removed from analysis if parents reported developmental disorders. Only one of the parents (mother or father) responded to the questionnaire. All parents were asked to report on a single child; all children involved were in confinement.

In total, 200 participants completed the survey. Of these, two were removed because parents reported on children aged 7 years or older; an additional five participants were removed for reporting developmental disorders (*n* = 3 autism, *n* = 1 sensory disorder, and *n* = 1 genetic disorder). The final sample, then, consisted of a total of 193 participants (all native and living in Portugal, except four participants who were non-native but with a self-reported good understanding of Portuguese). All respondents were parents (fathers *n* = 14; mothers *n* = 177), except 2, who were the main legal caregivers. As it was not mandatory to be only fathers or only mothers to answer the questionnaire, there is significantly lower participation of fathers when compared with the mothers. We found pieces of evidence (Grietens et al., 2004; Dave et al., 2008) that there is a high interparental agreement when reporting about problem behaviors in children, so we considered that the responses of both would have the same significance. The sample cannot be considered representative of a larger population.

### Procedures and Instruments

Before its dissemination, the survey was previously submitted and approved by the Ethics Review Board of the School of Health of the Polytechnic Institute of Porto. Before answering the questionnaire, all participants agreed to be involved in the study using an online informed consent form. The latter was formulated according to the World Medical Association Helsinki Declaration (2013), which contains the description and purpose of the study; an explanation that the data collected will be used for statistical purposes only; assurances that participation was voluntary; a statement that privacy and confidentiality of all data collected would be protected; and offering advice that the individual could withdraw from the study at any time without prejudice to them.

The survey was open online for about 1 month, from May 6 of 2020 to June 8 of 2020. It took approximately 20 min to be completed and included the following sections:

1. Sociodemographic data of parents and children; and conditions and changes related to confinement: as the personal situation changes [beginning of confinement, number of people living at home, monthly income, loss of job, changes in household income(s), and concerns about the COVID-19 pandemic] and home features (dimensions of the house, having a balcony at home, having a terrace at home, having a small garden, having a large outer space, or having no outer space). Parents’ perception about their confinement experience was asked using a five-point Likert-type scale (from “1–Very Negative” to “5–Very positive”) as well as their degree of concern about the situation of COVID-19 (from “1–Nothing worried” to “5–Very worried”). It is important to notice that in Portugal, the state of emergency was enacted on March 18 and ended on 2nd May. During this period, the population could only leave their homes to attend to basic needs, such as going shopping for food or going to the pharmacy. Outdoor activities, such as running or bike riding, were allowed, when alone, and solely for short distances. Children could play outdoor for short periods. Schools were closed, and all levels of education shifted to distance learning. On May 18, children younger than 3 years were allowed to go back to nurseries, and children from ages 3 to 6 years went back to kindergarteners after June 1. However, the outdoor restrictions remained.

2. Time and conditions of exposure of children to media and screens, including:

- Exposure time (in hours) to television (TV), computer, videogames, tablet, cellphone, and Internet, before and during the confinement, on weekdays and weekends, in a scale varying from 1 to 9 (1 = none, 2 = less than 1 h, 3 = 1 h, 4 = 2 h, 5 = 3 h, 6 = 4 h, 7 = 5 h, and 8 = more than 5 h). We computed a total score and a score for the difference between total media exposure during and before confinement.
- Parents’ perceptions about increased exposure to screens (TV, computer, video games, tablet, and cellphone), during the confinement, on a five-point Likert-type scale (from “1—Strongly Disagree” to “5—Totally Agree”). We computed a total score.
- Parents’ behavior while children used devices during the confinement, using a five-point Likert-type scale (1 = never, 2 = rarely, 3 = sometimes, 4 = very often, and 5 = always) on the following: “let children use the devices alone;” “sat close to the children but didn′t interact;” “talked with children about what they were doing or viewing;” and “participated with children when using devices.”

3. Behavioral and emotional symptoms on children during the confinement period, evaluated using the following scales:

- Baby Pediatric Symptom Checklist (BPSC) (Perrin et al., 2016), for children younger than 18 months.
- Preschool Pediatric Symptom Checklist (PPSC) (Perrin et al., 2016), for children from 18 to 66 months.

These are two components from the Survey of Well-Being of Young Children that assess behavioral and emotional symptoms for children younger than 18 months (BPSC) and from 18 to 66 months (PPSC). For each item on both scales’ response options are “Not at All,” “Somewhat,” and “Very Much.” The BPSC has 12 items, divided into three subscales (irritability, inflexibility, and difficulties with routines changings), each with four items. Subscale scores are determined by assigning a “0” for each “Not at All” response, a “1” for each “Somewhat” response, and a “2” for each “Very Much” response and then totaling the results. The range of the BPSC goes from 0 to 24, and any summed score of 3 or more on any of the three subscales indicates that the child is suspected of behavioral changes and needs further evaluation or investigation. The PPSC has 18 items divided into four domains of interest (“Internalizing,” “Externalizing,” “Attention Problems,” and “Parenting Challenges”). Internalizing items reflect the emotional or psychological state of the child, including depressive or anxiety symptoms. Externalizing items are related to the child reactions to other people or stressors, as hostility or aggression. Attention problems items ask about children’s ability to sustain focus or persistence in activities. Parenting challenges items reflect issues that parent’s face in raising their children. The total score can be assigned as on the BPSC, and a total score of 9 or greater, on a range of 0 to 36, indicates that a child is “at risk” of behavioral changes (Perrin et al., 2016; Moreira et al., 2019). There are two final questions in both surveys about parents’ concerns with the child’s learning/development and behavior. The Portuguese versions of these scales were used (Moreira et al., 2019).

## Results

### Descriptive Statistics and Relationship Between Children’s Emotional and Behavioral Problems and Age and Sex

The respondents were aged 22 to 50 years (mean = 36.44 *SD* = ± 4.35), mostly female (92.2%; *n* = 178)), and were the primary caregivers of children aged 6 to 82 months (mean = 42.86 *SD* = ± 20.65), mostly boys (56%, *n* = 108). The caregivers’ educational level was high: approximately 40.9% (*n* = 79) had a bachelor’s degree, and 35.2% (*n* = 68) had a masters’ degree or similar. Before the confinement due to the COVID-19 pandemic, the majority (84.5%, *n* = 163) had a full-time job, 8.8% (*n* = 17) had a part-time job, and 3.6% (*n* = 7) were unemployed. Most children had attended an educational establishment before the period of confinement (81.9%, *n* = 158). Of the parents, 83.9% (*n* = 162) responded to the PPSC test, and the other 16.1% (*n* = 31) responded to the BPSC, so 162 children were in the range of 18 months to 6 years and 12 months old (73 girls and 89 boys), and 31 (12 girls and 19 boys) were between 6 and 18 months old.

The majority of all the participant parents (71%, *n* = 137) agreed that exposure time to TV had increased during the confinement. Approximately 25% of the parents felt that children had increased the time using tablets (*n* = 49) and cellphones (*n* = 46) during the confinement. The majority of the parents (70.5%, *n* = 136) disagreed that exposure time to videogames has increased during the confinement period. Regarding computers, 59.1% (*n* = 114) disagreed that the use of these devices has increased during confinement (Table 1).

**TABLE 1:** Descriptive statistics of parents’ perceptions about increasing screen time exposure to children during confinement.

	Television		Videogames		Computer		Tablet		Cellphone	
	Freq.	%	Freq.	%	Freq.	%	Freq.	%	Freq.	%
Totally disagree	26	13.5	136	70.5	114	59.1	93	48.2	82	42.5
Disagree	15	7.8	13	6.7	13	6.7	17	8.8	19	9.8
Don’t agree or disagree	15	7.8	18	9.3	16	8.3	15	7.8	17	8.8
Agree	87	45.1	18	9.3	33	17.1	49	25.4	46	23.8
Totally agree	50	25.9	8	4.1	17	8.8	19	9.8	29	15.0
Mean ± *SD*	3.62 ± 1.31		1.70 ± 1.21		2.10 ± 1.47		2.40 ± 1.52		2.59 ± 1.58	

Independent samples *t*-test showed no differences between boys (*n* = 19) and girls (*n* = 12) (*t* = −0.150; *p* = 0.882) regarding BPSC Total and a trend toward significance regarding PPSC Total (*t* = −1.901; *p* = 0.059), with boys (*n* = 89) showing a higher mean than girls (*n* = 73) (*M* = 7.49; *M* = 5.89, respectively). Pearson’s correlation showed no significant correlation between age and child’s emotional and/or behavioral problems (Table 2).

**TABLE 2:** Relationship between children’s emotional and behavioral problems and age and sex (independent-samples *t*-test).

	BPSC irritability range_0–8	BPSC inflexibility range_0–8	BPSC routines range_0–8	BPSC total range_0–24	
Gender	Mean ± STD	Mean ± STD	Mean ± STD	Mean ± STD	
Girls (*n* = 12)	1.17 ± 1.26	2.25 ± 1.54	2.33 ± 2.31	5.75 ± 4.00	
Boys (*n* = 19)	2.11 ± 1.66	1.94 ± 1.47	1.89 ± 1.91	5.95 ± 3.27	
*T*	−1.669	0.547	0.574	−0.150	
*p*-value	0.106	0.588	0.570	0.882	

	**PPSC internalizing range 0–12**	**PPSC externalizing range 0–8**	**PPSC attention problems range 0–6**	**PPSC parenting challenges range 0–10**	**PPSC total range 0–36**
**Gender**	**Mean ± STD**	**Mean ± STD**	**Mean ± STD**	**Mean ± STD**	**Mean ± STD**

Girls (*n* = 73)	2.29 ± 1.49	0.51 ± 1.11	1.40 ± 1.60	1.70 ± 1.74	5.89 ± 4.61
Boys (*n* = 89)	2.33 ± 1.95	0.89 ± 1.19	1.80 ± 1.79	2.48 ± 2.38	7.49 ± 5.87
*T*	−0.138	−2.089	−1.486	−2.346	−1.901
*p*-value	0.891	0.038	0.139	0.020	0.059

### Relationship Between Children’s Emotional and Behavioral Problems and Variables Related to Confinement

For the analysis, we defined as dependent variables the total scores of BPSC and PPSC and used them for each independent variable related to confinement. Independent-samples *t*-tests were applied to the following independent variables: having a balcony at home, having a terrace at home, having a small garden at home, having a large outer space, having no outer space, parents having lost their job due to COVID-19, and parent teleworking. We used Pearson’s correlation between BPSC and PPSC total scores and the following independent variables related to confinement: number of people at home, monthly income, percentage of income loss, square meters of home, parents’ perception about confinement experience, concerning about COVID-19, and children’s number of days in confinement.

Regarding children from 6 to 18 months, independent-samples *t*-test showed no differences in BPSC between those reporting “yes” or “no” for the following variables related to the confinement condition: income loss, having a balcony at home, having a terrace at home, having a small garden, having a large outer space, having no outer space, parents having lost their job due to COVID-19, and parent teleworking (Table 3). There was no significant correlation between BPSC and the variables: number of people at home, monthly income, percent of income loss, house dimension in squared meters during the confinement, parents’ worries about COVID-19, and confinement experience. BPSC showed a negative correlation with the child’s number of days in confinement (*r* = −0.414, *p* = 0.021).

**TABLE 3:** Relationship between children’s emotional state and behavior and factors related to confinement (independent-samples *t*-test).

			Yes Mean ± *SD* (*n*)	No Mean ± *SD* (*n*)	Mean difference	*t*	*p*-value
	Income loss	BPSC total	6.25 ± 3.04(16)	5.47 ± 4.02(15)	0.783	0.615	0,544
		PPSC total	6.80 ± 5.65(91)	6.73 ± 5.07(71)	0.070	0.082	0,935
	Balcony at home	BPSC total	5.95 ± 2.94(20)	6.27 ± 4.50(11)	0.623	0.467	0,644
		PPSC total	6.97 ± 5.63(104)	6.41 ± 4.94(58)	–0.557	–0.630	0,530
Home features	Terrace at home	BPSC total	5.50 ± 2.59(6)	5.96 ± 3.74(25)	0.460	0.284	0,779
		PPSC total	6.95 ± 5.56(66)	6.65 ± 5.29(96)	–0.309	–0.357	0,721
	Small garden	BPSC total	3.83 ± 2.23(6)	6.36 ± 3.62(25)	2.527	1.627	0,115
		PPSC total	6.98 ± 5.44(50)	6.68 ± 5.39(112)	–0.301	–0.328	0,743
	Large outer space	BPSC total	5.86 ± 2.97(7)	5.88 ± 3.71(24)	0.018	0.012	0,991
		PPSC total	6.10 ± 4.80(48)	7.05 ± 5.61(114)	0.948	1.023	0,308
	No outer space	BPSC total	7.20 ± 5.72(5)	5.61 ± 3.02(26)	–1.585	–0.922	0,364
		PPSC total	8.06 ± 7.13(16)	6.63 ± 5.17(146)	–1.432	–1.010	0,314
	Lost job due to COVID-19	BPSC total	7.00 (1)	5.83 ± 3.56(30)	1.167	0.322	0,750
Personal situation		PPSC total	3.67 ± 3.06(3)	6.83 ± 5.41(159)	–3.163	–1.008	0,315
	Teleworking	BPSC total	5.85 ± 3.92(20)	5.91 ± 2.77(11)	–0.059	–0.44	0,965
		PPSC total	6.65 ± 5.33(96)	6.95 ± 5.50(66)	–0.309	–0.357	0,721

In the case of older children (from 18 months to 6 years old), independent-samples *t*-test showed no differences in PPSC between those reporting “yes” or “no” for the following variables related to the confinement condition: income loss, having a balcony at home, having a terrace at home, having a small garden, having a large outer space, having no outer space, parents having lost their job due to COVID-19, and parent teleworking (Table 3). Pearson’s correlation showed a negative correlation between PPSC and the parents’ perceptions about the confinement experience (*r* = −0.332; *p* = 0.00; Table 4). There was no significant correlation between PPSC and the following variables: number of people at home, monthly income, percent of income loss, house dimension in squared meters during the confinement, parents’ worries about COVID-19, confinement experience, and the child’s number of days in confinement (Table 4).

**TABLE 4:** Correlations between children’s emotional state and behavior and factors related to confinement period.

		*R*	
		BPSC total	PPSC total
Home features	Square meters home	0.065	0.023
	Number of people at home	0.250	0.105
	Monthly Income	0.031	–0.094
	% income loss	–0.301	0.184
Personal situation	Perception about confinement experience	–0.092	−0.332**
	Concerns about COVID-19	0.243	–0.44
	Children’s number of days in confinement	−0.414*	0.023

*p < 0.05. **p < 0.01.

### Relationship Between Children’s Emotional and Behavioral Problems and Exposure to Screen-Related Variables

Regarding PPSC domains, PPSC Attention Problems showed positive correlations with total hours of exposure to screens during confinement on weekdays (*r* = 0.288, *p* = 0.00) and total hours of exposure to screens during confinement, on weekends (*r* = 0.257, *p* = 0.001). Parenting Challenges showed positive correlations with total hours of exposure to screens during confinement on weekdays (*r* = 0.285, *p* = 0.000), total hours of exposure to screens during confinement, on weekends (*r* = 0.301, *p* = 0.000), and the difference between total exposure to screens during and before confinement, on weekends (*r* = 0.230, *p* = 0.003). A positive correlation was also found between total hours of exposure to screens during confinement, on weekends (*r* = 0.168, *p* = 0.032) and the difference between total exposure to screens during and before confinement, on weekends (*r* = 0.216, *p* = 0.006) and the PPSC Externalization scale (Table 5). We found positive correlations between the results of the PPSC Total and the following variables: total hours of exposure to screens during confinement on weekdays (*r* = 0.271, *p* = 0.000) and total hours of exposure to screens during confinement, on weekends (*r* = 0.266, *p* = 0.001). We also found a positive correlation between the total PPSC and the difference in the total hours of exposure during and before confinement on weekends (*r* = 0.195, *p* = 0.013) (Table 5).

**TABLE 5:** Correlations between children’s emotional state and behavior and exposure to screens.

*r*									
	Children less than 18 months (*n* = 31; 12 girls, 19 boys)				Children + 18 months to 6 years old (*n* = 162; 73 girls, 89 boys)				
	BPSC irritability	BPSC inflexibility	BPSC routines	BPSC total	PPSC internalizing	PPSC externalizing	PPSC attention problems	PPSC parenting challenges	PPSC total
Total hours of exposure to screens during confinement, on weekdays	–0.053	–0.197	0.037	–0.085	0.114	0.132	0.288**	0.285**	0.271**
Total hours of exposure to screens during confinement, on weekends	–0.015	–0.194	0.060	–0.054	0.084	0.168*	0.257**	0.301**	0.266**
Difference between total exposure to screens during and before confinement, on weekdays	–0.027	–0.101	−0, 138	–0.135	0.079	0.035	0.135	0.114	0.121
Difference between total exposure to screens during and before confinement, on weekends	0.112	0.037	–0.028	0.050	0.118	0.216**	0.056	0.230**	0.195*
Parent’s perception about the increasing of children screens exposure during confinement	0.209	–0.264	0.220	0.111	0.300**	0.235**	0.218**	0.302**	0.338**
Children use devices alone, during confinement	0.103	0.025	0.094	0.111	0.153	0.161*	0.071	0.091	0.144
Parents sit close to the child while using devices but did not interact, during confinement	–0.288	–0.005	–0.322	–0.319	–0.005	0.058	0.072	0.075	0.064
Parents talk about what children are doing or viewing when using devices, during confinement	0.071	0.100	–0.146	–0.011	–0102	–0.055	0.119	–0.056	–0.029
Parents participate with children when using devices, during confinement	0.018	–0.016	–0.067	–0.038	−0.264**	–0.032	–0.075	–0.136	−0.170*

*p < 0.05. **p < 0.01.

Parents’ perceptions about the increase of child’s media exposure during confinement was correlated to PPSC (*r* = 0.338, *p* = 0.00) and with its all domains: Internalization (*r* = 0.300, *p* = 0.000), Externalization (*r* = 0.235, *p* = 0.003), Attention Problems (*r* = 0.218, *p* = 0.005), and Parenting Challenges (*r* = 0.302, *p* = 0.000). The attitude of participation of the parents while children are using their devices during confinement was also associated to PPSC Total (*r* = −0.170, *p* = 0.030) and PPSC Internalization (*r* = −0.264, *p* = 0.001), whereas children using devices alone during confinement showed a positive correlation (*r* = 0.161, *p* = 0.041) with PPSC Externalization. All other factors have not shown any correlation with either BPCS Total or PPSC Total (*p* > 0.05) (Table 5).

## Discussion and Conclusion

The whole world has been affected by the COVID-19 pandemic and the confinement situation. Being confined, by itself, is an experience that can bring emotional and behavioral changes. The OECD (2020) warns that the stress and uncertainty associated with the COVID-19 outbreak potentially may have significant effects on children’s mental health. Furthermore, during the lockdown period, people, including children, were not able to do the amount of physical activity they used to do outdoors, especially if families lived in small apartments with no outer space, which could increase screen-time exposure and deepen psychological problems. In this exploratory study, we wanted to analyze the association between screen-time exposure during the lockdown period in Portugal due to the COVID-19 outbreak and the emotional and behavioral problems of infants and children aged younger than 7-years, with the BPSC and PPSC, while controlling for sociodemographic and confinement variables.

This is important because there is a trend toward increased screen exposure during confinement periods (Orgilés et al., 2020), and there is evidence that confinement is associated with behavior and emotional problems (Pearcey et al., 2020a). Given that there is evidence showing an association between screen-time exposure and behavioral and emotional problems (Özmert et al., 2002; Twenge and Campbell, 2018; Zhao et al., 2018), we hypothesized that greater screen-time exposure during the COVID-19 lockdown would lead children to have higher risks of emotional and behavioral problems. We found significant but weak correlations between these variables.

It should be noted that in our study, for children from 18 months to 6 years and 12 months, attention problems and parent challenges seem to be the most affected by the screen-time exposure. Surprisingly, internalization symptoms were not associated with screening exposure, despite pieces of evidence showing that screen-time exposure is related to reduced emotional regulation and self-control, poor sleep quality, less curiosity, and psychopathology, particularly anxiety and depression (Wu et al., 2015; Twenge and Campbell, 2018). Externalization symptoms were associated with exposure time during the confinement during weekends, which is in line with some reports that found higher aggressive behaviors in children with age-inappropriate viewing and higher duration screen viewing (Özmert et al., 2002; Conners-Burrow et al., 2011).

We did not find any consistent association between screen-time exposure and emotional or behavioral problems of infants aged 6 months to 18 months. Screen time seems to progressively increase with age, and well-being is more affected by screen-time exposure as children get older (Rosen et al., 2014; Twenge and Campbell, 2018). One explanation is that younger children may have spent more time watching age-appropriate TV or videos, whereas older children use more age-inappropriate videos or spend more time online. Another possibility is that older children have a steeper decrease in other fundamental activities when participating in screen activities, such as outdoors and free playtime activities. Some studies revealed that children suffered significant changes in their daily habits during the confinement period, spending more than 3 h using screens and halving the time spent in physical activities (Francisco et al., 2020; Morgül et al., 2020).

Focusing on aspects related to confinement, we hypothesized that confinement conditions could influence children’s emotional and behavioral states. Contrary to that and other reports (Cao et al., 2020; Pearcey et al., 2020b; Rodríguez-Rey et al., 2020), house conditions, household, and/or familiar income stability did not seem to have a major impact on the children’s well-being on this study. Maybe this is related to the fact that, in Portugal, children could still go outside to practice leisure activities, even if for short periods of time. However, the parents’ perceptions of their confinement experience proved to be important in the children’s behavior and emotional adjustment showing a bigger correlation to PPSC than screen exposure variables. Thus, when parents have a bad perception about their confinement experience, they tend to report more emotional and behavioral problems in their children from 18 months to 6 years and 12 months. In previous studies, parents who reported a greater impact of COVID-19 also reported higher levels of parental stress, associated with the increased use of harsh parenting and less parent–child relationship closeness and more emotional problems in their children (Chung et al., 2020; Orgilés et al., 2020). Also, we found a negative association between the number of days in confinement and the behavioral and emotional state of younger children (from 6 to 18 months), contrary to the evidence that longer quarantine periods are associated with poorer psychological outcomes (Brooks et al., 2020). This may reinforce the hypothesis that an increased presence of parents may improve emotional and behavioral outcomes in young children.

Moreover, when parents report participating with their children while using devices, there are fewer emotional and behavioral problems, and exteriorization problems increase in children when using devices alone. This supports the importance of co-viewing when children are exposed to screens, as the presence of a co-viewing parent is sufficient to alter children’s psychophysiological responses to media content, facilitates the learning process with “scaffolding,” and help children understand on-screen content (DeLoache et al., 2010; Council On Communications And Media, 2016; Rasmussen et al., 2017; Gottschalk, 2019). These findings highlight the importance of “doing together” as the gold standard for parents when children use technologies.

This study has several important limitations, including sample size, the lack of data before the confinement, and the bias associated with the subjective indirect measures with self-reporting data. The snowball distribution of the questionnaires proved to be a limitation for the study, as it did not allow to control the size of the sample for the different variables, so we could not equally distribute the groups for the data analysis. We chose to analyze data according to the age ranges defined by the BPSC and the PPSC, which further reinforced the small power sample for data analysis, so results have to be read and understood with careful attention. Also, BPSC and PPSC are screening scales, which may not be refined enough to capture the whole picture of children’s emotional or behavioral problems, as it would happen if we were using a fully comprehensive development assessment. Furthermore, most of our measures are subjective, self-reported by parents, and based on estimations. Thus, answers may also be influenced by social desirability bias. We also did not collect data regarding time spent by children in other activities, such as physical or outdoor activities, during the lockdown period due to COVID-19. This would have given valuable data regarding the effect of screen-time exposure during the COVID-19 confinement on lifestyle and fitness at younger ages.

However, showing a negative association between screen time and emotional and behavioral well-being among children during the lockdown period and highlighting the potential role of parents in children’s behavioral and emotional adjustment, the results of the study are important so that in future periods of confinement, parents seek to mediate the time and the conditions children are exposed to screens, to minimize their impact.

## Data Availability Statement

The raw data supporting the conclusions of this article will be made available by the authors, without undue reservation.

## Ethics Statement

The studies involving human participants were reviewed and approved by Ethics Review Board of the School of Health of the Polytechnic of Porto. The patients/participants provided their written informed consent to participate in this study.

## Author Contributions

RM, SF, and NR: conceptualization, methodology, and writing (review and editing). RM: data collection and original draft. RM and NR: data analysis. All authors have read and agreed to the submitted version of the manuscript.

## Conflict of Interest

The authors declare that the research was conducted in the absence of any commercial or financial relationships that could be construed as a potential conflict of interest.
